# Effectiveness of the Educational Technology “Sophia’s Story” in Transitional Care and Stimulation of Preterm Infants

**DOI:** 10.1590/0034-7167-2024-0393

**Published:** 2025-08-22

**Authors:** Jamile Gregorio Morelo, Pamela Brustolini Oliveira Rena, Rayla Amaral Lemos, Nádia Proença de Melo, Maria de La Ó Ramallo Veríssimo

**Affiliations:** I Centro Universitário Senac, Unidade Tiradentes. São Paulo, São Paulo, Brazil; II Universidade Federal de Viçosa. Viçosa, Minas Gerais, Brazil; III Universidade Federal de Juiz de Fora. Juiz de Fora, Minas Gerais, Brazil; IV Universidade de São Paulo. São Paulo, São Paulo, Brazil

**Keywords:** Transitional Care, Preterm Newborn, Child Development, Neonatal Nursing, Pragmatic Clinical Trial, Cuidado Transicional, Recién Nacido Prematuro, Desarrollo Infantil, Enfermería Neonatal, Ensayo Clínico Pragmático

## Abstract

**Objectives:**

to evaluate the effectiveness of the educational technology “Sophia’s Story: Battles and Triumphs of the Family in the Care and Development of the Premature Child” in promoting home-based stimulation for preterm infants and to assess associations between family, demographic, and behavioral factors and the promotion of home-based stimulation for preterm infants.

**Methods:**

a pragmatic, non-randomized clinical trial was conducted with 35 preterm infants discharged from the neonatal intensive care unit in each group, approved by a Research Ethics Committee. Caregivers received standardized hospital discharge guidance, with the educational technology provided to the experimental group. Home-based stimulation was assessed using the Infant-Toddler Home.

**Results:**

the experimental group obtained higher scores in the overall mean (p=0.001) and in the following subscales: absence of punishment and restriction (p=0.005); availability of materials, toys, and appropriate games (p=0.001); maternal involvement with the child (p=0.001); and opportunity for variation in daily stimulation (p=0.025).

**Conclusions:**

exposure to the educational technology resulted in greater home-based stimulation, supporting transitional care.

## RESUMEN

**Objetivos:**

evaluar la efectividad de la tecnología educativa “Historia de Sofía: batallas y conquistas de la familia en el cuidado y desarrollo del niño prematuro” para la promoción de estímulos domiciliarios para lactantes pretérmino; verificar asociaciones entre factores familiares, demográficos y conductuales y la promoción de estímulos domiciliarios para lactantes pretérmino.

**Métodos:**

ensayo clínico pragmático, no aleatorizado, con 35 lactantes egresados de la unidad de cuidados intensivos neonatales en cada grupo, aprobado por el Comité de Ética en Investigación. Los responsables recibieron orientación estandarizada de alta hospitalaria, con la tecnología añadida al grupo experimental. La estimulación domiciliaria se evaluó con el instrumento *Infant-Toddler Home*.

**Resultados:**

el grupo experimental obtuvo puntuaciones superiores en la media general (p=0,001) y en las siguientes subescalas: ausencia de castigo y restricción (p=0,005); disponibilidad de materiales, juguetes y juegos apropiados (p=0,001); implicación materna con el niño (p=0,001); y oportunidad de variación en la estimulación diaria (p=0,025).

**Conclusiones:**

el contacto con la tecnología proporcionó más estímulos domiciliarios, apoyando el cuidado transicional.

## INTRODUCTION

In recent decades, the global reduction in infant mortality has been significant, and neonatal mortality has become the primary component of infant mortality in many regions, including Brazil. One of the main causes of neonatal mortality is preterm birth, which accounts for 11% of deliveries, with an incidence ranging from 5% to 18%, depending on the location^(1,2)^. Preterm birth is defined as delivery occurring before 37 weeks of gestation^(3)^.

In addition to being a risk factor for mortality, this condition can lead to impairments in various aspects of health and child development, affecting skill acquisition due to the immaturity of organs and physiological systems^(2)^.

Given the magnitude of this issue, two Sustainable Development Goals (SDGs) aim to address this situation. Goal 3 proposes eliminating preventable deaths in this age group by 2030^(1)^, which can be achieved through perinatal care that ensures better birth conditions. Goal 4 focuses on guaranteeing access to quality early childhood development^(4)^, which involves providing care and opportunities that extend beyond mere survival.

A specific aspect of care for preterm infants is the involvement of multiple healthcare professionals. This care is generally focused on intensive support for adaptation to extrauterine life and may continue even after hospital discharge if there is a high clinical risk or if the child’s health condition requires daily specialized care^(5)^.

In this context of intensive care, parents who accompany their infants during hospitalization report feeling insecure about the care they will need to provide afterward. According to them, the healthcare team does not prepare them for discharge as they would like, considering all the complexities of prematurity^(6)^. Thus, throughout hospitalization, it is essential to prepare the family for discharge with interventions that support, guide, and, most importantly, empower parents for home care^(7)^.

This preparation is crucial for two reasons. The first concerns the interaction between the infant and the family, a central aspect of child development^(8)^. The second relates to parental insecurity regarding infant care, as the lack of preparation during hospitalization for the transition to home care weakens actions that support development^(9)^.

The lack of guidance and support in transitional care, as reported by parents of preterm infants, can hinder these children’s development by exposing them to less stimulating environments^(10)^. Furthermore, child development is often inadequately monitored in healthcare services, which tend to prioritize growth parameters such as weight gain and height over functional skills and stimulation-oriented care^(11)^. Therefore, the transition from hospital to home care represents a critical period of adaptation and reorganization of family routines to meet the comprehensive needs of these infants, extending beyond concerns about anthropometric data and signs of illness^(12)^.

To assist parents in this transition process and during the early years of life, promoting child development, an educational resource was created for families of preterm infants: *Sophia’s Story: Battles and Triumphs of the Family in the Care and Development of the Premature Child* ^(13)^. This educational technology (ET) is a book designed to facilitate access to knowledge about infant care and development before and after hospital discharge, starting from the neonatal intensive care unit (NICU). It provides guidelines for care and developmental promotion, featuring simple texts, numerous illustrations, and a chart detailing functional skills—such as mobility acquisition, social interaction, and self-care—for children aged 0 to 3.5 years^(13)^.

It addresses this topic by emphasizing the importance of interactions and affection, illustrating how interaction and caregiving processes, when experienced in welcoming and stimulating environments, enhance child development through simple texts and numerous illustrations^(13)^.

Development-promoting care is vital for preterm children, as previously described^(6-10)^. Additionally, investing in child development is an effective strategy for a country to eliminate extreme poverty, promote inclusive economic growth, and expand equal opportunities, which is why this topic is included in the SDGs^(14)^.

Thus, the research question in this study was: What are the effects of the ET *Sophia’s Story* on home-based stimulation of preterm infants? What other variables influence home-based stimulation of preterm infants?

Investigating the effects of this ET is justified by the significant impact of preterm birth on child development and the frequent lack of specific guidance in transitional care, which may result in insufficient or inadequate stimulation of these children at home. Understanding the effects of applying this technology, along with other variables that may influence home care in the context of prematurity, will contribute to validating this ET as a resource for transitional care.

## OBJECTIVES

- To evaluate the effectiveness of the ET *Sophia’s Story: Battles and Triumphs of the Family in the Care and Development of the Premature Child* in promoting home-based stimulation for preterm infants.
- To verify associations between family, demographic, and behavioral factors and the promotion of home-based stimulation for preterm infants.

## METHODS

### Ethical aspects

The study was approved by the ethics committees of the participating educational and healthcare institutions, following Resolution No. 466/12 of the Brazilian National Health Council. It was also registered on the virtual platform of the Brazilian Registry of Clinical Trials under the primary identifier RBR-2SK64P. Additionally, the study adhered to CONSORT recommendations to ensure transparency and accuracy in reporting results.

The intervention utilized an ET that can be considered reliable and safe in addressing the topic, given the robust methodology adopted for its development^(11)^ and the validation of its content by professionals and families (data still in the process of publication). This study is part of the clinical trials required to assess the ET’s potential effects on improving family care and promoting child development.

### Study design, period, and location

This was a pragmatic, non-randomized clinical trial (PCT) in which the independent variable was an educational intervention, and the expected outcome was an increased provision of home-based stimulation to promote the development of preterm infants, specifically concerning family interaction with the baby and the characteristics and opportunities of the environment that favor child development^(7,8)^.

PCTs are conducted under less strict conditions than explanatory clinical trials, as their purpose is to assess the effectiveness of an intervention in contexts that replicate real-world clinical practice. Thus, they allow the study to adapt to the needs and characteristics of the participants. Another relevant aspect of the study design concerns the verification of intervention effectiveness, defined as the assessment of its effects in a specific sample or population under uncontrolled conditions, that is, in a “real-world” setting^(15)^.

Non-randomization was considered necessary due to the impossibility of forming the groups simultaneously, as the families of newborns had significant contact with each other during hospitalization in the NICU. Thus, the CG was composed of families whose babies had already been discharged at the beginning of data collection, while the experimental group (EG) consisted of families whose babies were hospitalized from that date onward (May 2018). This strategy ensured that families did not have the opportunity to exchange information about the ET.

Data collection was conducted through home visits to preterm infants born in a private general hospital in the eastern zone of São Paulo, where the ET was presented and offered.

### Population, sample, and inclusion and exclusion criteria

The study was conducted with dyads of parents and children born at the study site. The sample size was determined to ensure that the standardized difference between groups in the assessment instrument scores would be statistically significant in an independent sample test, defined as 80 dyads, with 40 assigned to the CG and 40 to the EG.

To form the CG, a review of the NICU admission records was conducted to identify preterm newborns between January and May 2018 who had already been discharged from the hospital. In the EG, preterm newborns who were discharged after May 2018 were prospectively included. Both groups received the same standardized discharge guidance. Additionally, in the EG, the ET was presented and provided. After offering the book, the researcher requested authorization for telephone contact within 30 days to confirm interest in participating in the study and to schedule a home visit.

Dyads of parents and infants were eligible for the study if they met the following conditions: infants born at any level of prematurity, regardless of other medical conditions; parents with the cognitive ability to read the educational material and who were responsible for the child’s usual care. Dyads were excluded if it was not possible to schedule a home visit after three attempts to contact them by phone at different times.

### Study protocol

The independent variable in the study was exposure to the ET at the time of hospital discharge guidance, and the outcome was the level of home-based stimulation provided to the infant, measured using the *Infant-Toddler Home* (IT-HOME) instrument^(16)^.

The IT-HOME was developed to assess, in a naturalistic context, the quality and quantity of stimulation and support available in the home environment for the child, who is understood as an active recipient of objects, events, and transactions occurring within the family environment^(17)^. It is widely used by researchers in many countries to evaluate the effects of parenting interventions and child development promotion, particularly among children in vulnerable situations^(18)^. The instrument consists of 45 items, either observed or reported, related to the contextual and organizational characteristics of the home environment that influence child development, divided into six subscales^(16)^:

1. Maternal verbal and emotional responsiveness: assesses communicative and affective interactions between the caregiver and the child.
2. Absence of restriction and punishment: examines the methods used by the caregiver to discipline the child.
3. Organization of the physical and temporal environment: evaluates the child’s routine within the family context and the spaces and objects designated for the child.
4. Availability of materials, toys, and appropriate games: identifies and categorizes, according to the child’s age, the variety and types of toys available.
5. Parental involvement with the child: measures the quality and quantity of interaction the child receives from the primary caregiver.
6. Opportunity for variation in daily stimulation: assesses the opportunities for social interactions offered to the child beyond contact with the mother.

All items in each subscale are scored as YES or NO, according to a manual that provides an explanation for each item and examples for scoring. Higher scores indicate a more stimulus-enriched family environment, while lower scores, in the bottom quartile, represent a risk for child development^(16,17)^. Thus, the instrument was chosen for its ability to demonstrate whether families in the EG provided a more favorable environment for child development compared to families in the CG, based on the hypothesis that differences in results between the groups would be associated with access to the ET.

The principal researcher was responsible for administering the IT-HOME through observation and a semi-structured interview with the child’s primary caregiver. The presence of the infant was required to obtain immediate information on interaction patterns between the caregiver and the child. The complete assessment took approximately one hour.

Data collection was conducted through home visits scheduled in advance by phone between May and November 2018. During these visits, the researcher was accompanied by one of three assistants. The assistants were nurses with experience in the care of preterm infants and acted as observers, as required by the data collection instrument. They received training on the psychometric parameters of the IT-HOME^(16)^. There was no blinding of researchers or families, as the principal researcher participated in all data collection and was also the person who introduced the ET at the time of hospital discharge. All participants had the opportunity to review the informed consent document and signed the Informed Consent Form (ICF).

To collect data on family, demographic, and behavioral factors, as well as characteristics of preterm infants that could influence their development, a structured questionnaire was used. It covered topics such as pregnancy planning, preparation for the baby’s arrival, reading materials on child development, reports of positive or negative aspects related to care, the occupation of primary caregivers, and years of education. These data were obtained through responses to the questionnaire, including questions such as: “Was there preparation for the baby’s arrival?” with two possible answers: Yes or No. All this information, particularly that related to preparation for the baby’s arrival, could influence the IT-HOME results by enhancing caregivers’ understanding of the child’s needs.

To verify homogeneity between groups, data were collected on the infants’ physical and physiological characteristics at birth, as well as post-discharge referrals. Following this, the IT-HOME was administered.

Data were recorded during home visits using a printed instrument and later entered twice into Excel^®^ version 2016 spreadsheets to ensure accuracy.

### Analysis of results and statistics

To assess the effectiveness of the intervention with the ET *Sophia’s Story*, a statistical analysis was conducted to compare the groups and examine the association between IT-HOME scores and the variables of interest. For the comparison of numerical variables, *t-tests*, Wilcoxon-Mann-Whitney, Brunner-Munzel, and ANOVA tests were used, as well as the Kruskal-Wallis test in cases involving multiple groups. For the comparison of categorical variables, chi-square tests, Fisher’s exact test, and Spearman’s coefficient were applied.

## RESULTS

**Figure 1:**
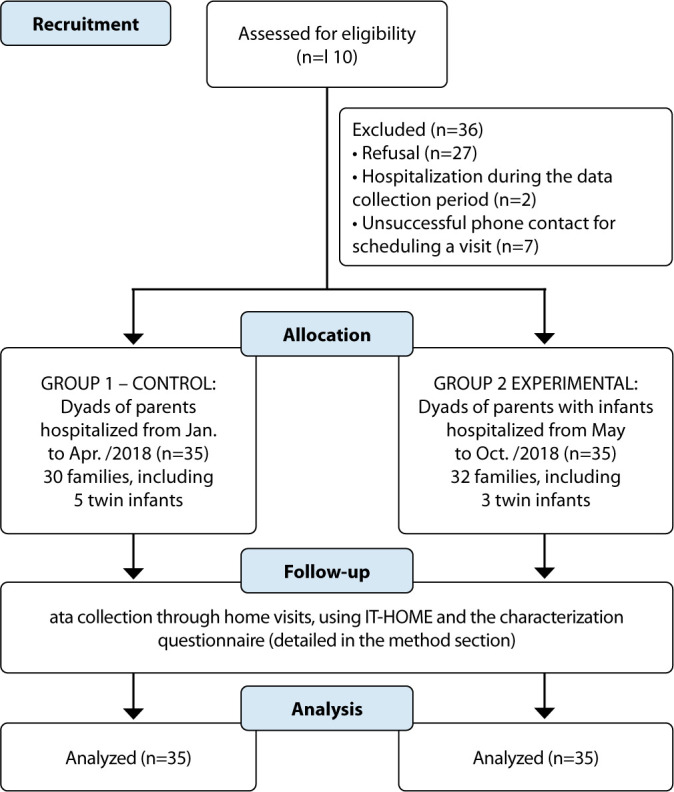
Flowchart of the dyads participating in the study, including recruitment, allocation, and application of data collection instruments, São Paulo, São Paulo, Brazil, 2019

The flowchart of the families participating in the study, including recruitment, allocation into the control and EG, and the application of data collection instruments, is presented in Figure 1.

Home visits lasted between 40 and 120 minutes, with an average duration of 80 minutes for most families (n=52, 74.3%). The CG comprised 30 families, while the EG included 32; in total, the two groups consisted of 35 infants, as twin infants were included. The decision was made to retain twins, even though the characteristics of family and environmental variables were similar, since, when applying the data collection instrument, different scores were observed between the children regarding environmental stimulation in both groups.

All infants had their mother as the primary caregiver; the majority (41 - 58.5%) had a secondary caregiver: the father (32 - 78%), the grandmother (5 - 12.3%), or a nanny (4 - 9.7%). On average, mothers in the CG had 15.8 years of education, and the secondary caregiver had 13.3 years; in the EG, mothers had 16 years of education, while the secondary caregiver had an average of 14.2 years. Regarding the educational background of primary caregivers, the majority had training in the humanities, with 18 (51.4%) in the CG and 17 (48.6%) in the EG. No statistically significant difference was observed between the groups.

Regarding the infants’ age at the time of data collection, there was a statistically significant difference between the groups (p<0.001 - Wilcoxon-Mann-Whitney test), with the corrected mean age being 3.2 months in the CG and 1.26 months in the EG. As for the birth conditions of the infants, a variable considered for group homogeneity, no significant differences were found (Table 1).

**Table 1:** Physical and physiological characteristics of newborns in the control (CG) and experimental (EG) groups at birth, São Paulo, São Paulo, Brazil, 2019

	CG (Control Group)	EG (Experimental Group)	*p* value
Birth Conditions			
Mean Gestational Age	34.4 weeks	34.4 weeks	0.715**
Apgar Score at 1 Minute	7.7 points	7.8 points	0.290**
Apgar Score at 5 Minutes	8.8 points	9.0 points	0.640**
Mean Birth Weight	2.2 grams	2.1 grams	0.384***
Weight/Gestational Age			
AGA* (Appropriate for Gestational Age)	25 (71.2%)	27 (77.2%)	0.785****
SGA* (Small for Gestational Age)	09 (25.7%)	08 (22.8%)	
LGA* (Large for Gestational Age)	01 (2.8%)	0 (0.0%)	

* *AGA – Appropriate for Gestational Age; SGA – Small for Gestational Age; LGA – Large for Gestational Age;*

** *Wilcoxon-Mann-Whitney test;*

*** *Two Sample t-test;*

**** *Fisher’s Exact Test.*

Regarding the medical diagnoses of newborns during hospitalization, the most common condition was jaundice, with 26 cases (74.2%) in the CG and 28 cases (80%) in the EG, with no significant differences between the groups. No differences were found between infants in the CG and EG regarding post-discharge referrals, indicating similar complexity of care between the groups.

Concerning the application of IT-HOME, the EG achieved higher average scores, both in the total instrument score and in most subscales (Table 2), indicating greater environmental stimulation for infants in the group that had access to the ET.

**Table 2:** Distribution of the mean IT-HOME scores in the control (CG) and experimental (EG) groups, São Paulo, São Paulo, Brazil, 2019.

Subscales (Variables)	Mean Score per Subscale		*p* value
	CG (Control Group)	EG (Experimental Group)	
Maternal Emotional and Verbal Responsiveness	9.54	9.60	0.513*
Absence of Punishment and Restriction	4.46	5.03	0.005*
Organization of the Physical and Temporal Environment	4.37	4.40	0.836*
Availability of Materials, Toys, and Appropriate Games	3.03	4.14	0.001*
Maternal Involvement with the Child	2.37	3.80	<0.001*
Opportunity for Variation in Daily Stimulation	2.80	3.26	0.025*
***IT-HOME Total Score***	26.57	30.23	<0.001**

* *Wilcoxon-Mann-Whitney test;*

** *Two Sample t-test.*

Family behavioral characteristics were associated with IT-HOME results. There was homogeneity between the groups regarding these characteristics, as described below.

The majority of families in both groups reported more positive than negative aspects regarding infant care (60 - 85.7%). These reports were significantly associated with the *Maternal Emotional and Verbal Responsiveness* subscale, with a mean score of 9.8 and a median of 10 points, whereas reports with more negative aspects, or both negative and positive aspects (10 - 14.3%), had a mean score of 8.2 and a median of 8 points (p-value <0.01 – Brunner-Munzel test). This same variable also influenced the total IT-HOME score, with an average score of 28.8 and a median of 29, compared to a mean of 29 and a median of 25 in cases where more negative reports were present (p-value = 0.007 - Two Sample t-test).

The report of reading specific materials on child development (CD) as a form of preparation for the baby’s arrival had a statistically significant influence on two subscales and the total IT-HOME score, occurring in 16 families (22.8%), distributed across both groups. In the *Availability of Materials, Toys, and Appropriate Games* subscale, the dyads in this group had a mean score of 4.31 and a median of 4.5, compared to a mean of 3.37 and a median of 3 in the absence of such readings (p-value = 0.019 - Wilcoxon-Mann-Whitney test). In the *Maternal Involvement with the Child* subscale, the mean score was 3.63 and the median was 3.5, compared to a mean of 2.93 and a median of 3 for those who did not read (p-value = 0.043 - Wilcoxon-Mann-Whitney test). The mean total IT-HOME score for families with this positive variable was 30.1, with a median of 31, compared to a mean of 27.8 and a median of 28 for those who responded negatively (p-value = 0.029 - Two Sample t-test).

Only seven families (10%) attended a preparatory course for the baby’s arrival, three in the EG and four in the CG, and this variable influenced two subscales and the total instrument score. In the *Availability of Materials, Toys, and Appropriate Games* subscale, the dyads whose parents took the courses had a mean score of 4.71 and a median of 5, compared to a mean of 3.46 and a median of 3 for those whose parents did not attend courses (63 - 90%) (p-value = 0.026 - Wilcoxon-Mann-Whitney test). In the *Maternal Involvement with the Child* subscale, the mean score was 4.14 and the median was 4, compared to a mean of 2.97 and a median of 3 for those who did not take such courses (p-value = 0.026 - Wilcoxon-Mann-Whitney test). The total IT-HOME score for dyads whose families participated in courses was higher, with a mean of 31 and a median of 31, compared to a mean of 28.1 and a median of 28 for the others (p-value = 0.05 - Two Sample t-test).

Using the Basic Health Unit was reported by 52 families (74.2%) as a source of support for infant care, and this variable influenced two subscales. In the *Availability of Materials, Toys, and Appropriate Games* subscale, the mean score was 3.33 and the median was 3, higher than among families who did not use this resource, whose mean score was 2.39 and median was 3 (p-value = 0.012 - Wilcoxon-Mann-Whitney test). The *Maternal Involvement with the Child* subscale had a mean score of 3.33 and a median of 3 among families who used the UBS, compared to a mean score of 2.39 and a median of 3 among those who did not use this service (p-value = 0.027 - Wilcoxon-Mann-Whitney test). Regarding the total IT-HOME score, families who used the UBS had a mean score of 28.9 and a median of 26, while the others had a mean score of 26.7 and a median of 29 (p-value = 0.027 - Two Sample t-test).

The majority of families, 41 (58.6%), similarly distributed between the groups, reported engaging in enjoyable activities with the baby. These families had higher scores in the *Availability of Materials, Toys, and Appropriate Games* subscale, with a mean score of 3.39 and a median of 3, compared to a mean score of 2.39 and a median of 3 among those who did not engage in such activities (p-value = 0.027 - Wilcoxon-Mann-Whitney test). Engaging in such activities with the infant also influenced the *Maternal Involvement with the Child* subscale, with a mean score of 3.39 and a median of 4, compared to a mean score of 2.66 and a median of 3 among those who responded negatively (p-value = 0.027 - Wilcoxon-Mann-Whitney test). There was a difference in the mean total IT-HOME score between families who engaged in enjoyable activities with the baby, with a mean score of 29.32 and a median of 27.1, while the others had a mean score of 27.1 and a median of 26 (p-value = 0.027 - Two Sample t-test).

The subscales most influenced by family behavioral variables were *Availability of Materials, Toys, and Appropriate Games*, with five associated variables, and *Maternal Emotional and Verbal Responsiveness* and *Maternal Involvement with the Child*, with three associated variables each.

Additionally, beyond addressing the established objectives, more than half of the EG participants (18 - 56.2%) spontaneously provided comments on the ET, which were predominantly positive: they stated that they liked the ET (16 - 89%) and that they used it as an information source (14 - 78%).

## DISCUSSION

The ET proved effective in supporting families in providing a home environment with greater stimulation for child development. The absence of significant differences between groups regarding demographic and behavioral factors prior to the birth of the infants, as well as in the physical and physiological characteristics of the newborns and postnatal variables such as UBS use and engagement in enjoyable activities with the infants, suggests that the observed effects are potentially associated with the intervention, which was access to the ET.

Additionally, the lack of significant differences in parental occupation, education level, primary caregiver, birth characteristics, medical diagnoses, and post-discharge referrals of the infants between groups indicates that the strategy used to form the groups allowed for comparability.

The corrected age variable was the only one that showed a difference between groups, with a higher mean in the CG, which could have led to more favorable results for this group. This statement is supported by the understanding that interactions with infants increase as they grow and remain awake for longer periods^(19)^. By the end of the third month, an age already reached by the CG infants, they exhibit greater responsiveness and social engagement capacity^(20)^, which could have resulted in greater stimulation in this group, but this was not observed. Thus, the higher environmental stimulation scores may, in fact, be associated with a better understanding by families of its importance, reinforcing the positive effect of the ET.

The availability and variety of age-appropriate toys in the infants’ environment were greater in the EG compared to the CG, an aspect extensively covered in the ET^(13)^. Environments that provide toys for infants become more engaging for learning and stimulate development^(21)^.

The ET includes a chapter, “A Conversation About the Amazing World of Development,” which highlights the importance of toys and how they promote development, even for preterm and very young infants^(13)^. Families often do not consider playing and interacting with the baby necessary during the first months of life.

Parental practices and social interactions are also critical modulators of child development^(22)^. The exposure of EG families to the ET encouraged these interactions, leading them to become more engaged with their children and to provide opportunities for social interactions beyond contact with the mother. These themes are emphasized in the ET, as interaction through conversation during routine care, as well as engagement with other family and community members, is essential for child development^(13)^. On the other hand, CG infants received less environmental stimulation, as indicated by the *Restriction and Punishment* subscale of the IT-HOME.

A significant portion of families demonstrated availability and engagement with their baby even before birth through participation in preparatory courses for future caregiving and by reading materials on child development, characteristics that positively influenced IT-HOME results.

These characteristics align with the findings of a review that identified key points discussed in the literature regarding the influence of environmental context quality on child development. Positive interactions within an individual’s context, especially within the family, parental care, access to goods and services, and intrinsic factors of that environment are fundamental to child development^(23)^.

Two subscales showed no significant difference between the groups: *Maternal Emotional and Verbal Responsiveness* and *Organization of the Physical and Temporal Environment*, both of which indicate positive aspects of good caregiving in both groups. In the case of the first, most families were developing an emotional bond with their children, while the second may be associated with standardized discharge guidance provided by the healthcare service. This is because physicians recommend that parents avoid outings and exposing the baby to crowded enclosed environments, at least until the infant reaches two months post-discharge or obtains clearance from a pediatrician.

The results indicate that this ET assisted families in home care for preterm infants, making it an important resource for hospital discharge preparation for healthcare teams. This finding aligns with clinical trials of other ETs, which are considered useful for disseminating knowledge and information and, most importantly, facilitating communication^(24,25)^.

The investigation of parental interventions in real-world environments has been a key focus, particularly in evaluating the effectiveness of tools that support child development promotion^(26)^. Therefore, the ET *Sophia’s Story* can be considered an effective technology in achieving SDG target 4.2, as it enables parents of preterm infants to provide quality developmental stimulation for their children.

### Study limitations

The primary limitations of this study include the lack of randomization and blinding of both the participating families and the researcher, which may have positively influenced some families’ outcomes regarding child development. The absence of randomization was justified in the methodology and ensured the appropriate composition of the groups. Additionally, similarities in individual and family characteristics between the groups were observed, which can be considered a mitigating factor. Regarding the lack of blinding, the presence of observers and discussions of data collection records also helped minimize the potential for bias.

### Contributions to the Fields of Nursing, Health, and Public Policy

This study presents the ET *Sophia’s Story* as an important tool to support healthcare professionals in transitional care, as it contributed to redefining caregiving practices, fostering autonomy in child development^(27)^, and reinforcing the value of this resource in achieving SDG target 4.2.

## CONCLUSIONS

The exposure of families with preterm infants to the ET during hospital discharge preparation resulted in a greater provision of developmental stimuli in the home environment. This finding reinforces the validity of systematic educational interventions for the families of these children, supporting the Sustainable Development Goals agenda, particularly in relation to quality early childhood development.

Future research on the use of this ET, involving the follow-up of dyads and assessments of infant development at later ages, may contribute to a better understanding of the scope and impact of this resource.
