# Supplementary material for: Effectiveness of the Educational Technology “Sophia’s Story” in Transitional Care and Stimulation of Preterm Infants
Source: Rev Bras Enferm. 2025 Aug 22;78(Suppl 1):e20240393. doi: 10.1590/0034-7167-2024-0393 (PMC12380421; doi:10.1590/0034-7167-2024-0393)
Supplement: Translation

# Translation: Efetividade da tecnologia educativa “História de Sofia” no cuidado transicional e estimulação do lactente pré-termo

**DOI:** 10.1590/0034-7167-2024-0393pt

**Authors:** Jamile Gregorio Morelo, Pamela Brustolini Oliveira Rena, Rayla Amaral Lemos, Nádia Proença de Melo, Maria de La Ó Ramallo Veríssimo

**Affiliations:** I Centro Universitário Senac, Unidade Tiradentes. São Paulo, São Paulo, Brazil; II Universidade Federal de Viçosa. Viçosa, Minas Gerais, Brazil; III Universidade Federal de Juiz de Fora. Juiz de Fora, Minas Gerais, Brazil; IV Universidade de São Paulo. São Paulo, São Paulo, Brazil

**Keywords:** Cuidado Transicional, Recém-Nascido Prematuro, Desenvolvimento Infantil, Enfermagem Neonatal, Ensaio Clínico Pragmático

## Abstract

**Objetivos:**

avaliar a efetividade da tecnologia educativa “História de Sofia: batalhas e conquistas da família no cuidado e desenvolvimento da criança prematura” para a promoção de estímulos domiciliares para lactentes pré-termo; verificar associações entre fatores familiares, demográficos e comportamentais e a promoção de estímulos domiciliares para lactentes pré-termo.

**Métodos:**

ensaio clínico pragmático, não randomizado, com 35 lactentes egressos da unidade intensiva neonatal em cada grupo, aprovado por Comitê de Ética em Pesquisa. Os responsáveis receberam orientação de alta padronizada, adicionada da tecnologia ao grupo experimental. A estimulação domiciliar foi avaliada com o *Infant-Toddler Home*.

**Resultados:**

o grupo experimental obteve escores superiores na média geral (p=0,001) e nas subescalas: ausência de punição e restrição (p=0,005); disponibilidade de materiais, brinquedos e jogos apropriados (p=0,001); envolvimento materno com a criança (p=0,001); oportunidade de variação na estimulação diária (p=0,025).

**Conclusões:**

o contato com a tecnologia proporcionou mais estímulos domiciliares, apoiando o cuidado transicional.

## INTRODUÇÃO

Nas últimas décadas, a redução da mortalidade infantil em todo o mundo foi expressiva, e a mortalidade neonatal passou a representar a principal parcela da mortalidade infantil em muitas localidades, como no caso do Brasil. Uma das principais razões da mortalidade neonatal é o nascimento prematuro, que corresponde a 11% dos partos, com incidência de 5% a 18%, conforme a localidade^(1,2)^. O nascimento prematuro é definido como aquele que acontece antes das 37 semanas de gestação^(3)^.

Além de ser um fator de risco para a mortalidade, essa condição pode acarretar prejuízos em diversas dimensões da saúde e do desenvolvimento infantil, interferindo na aquisição de habilidades devido à imaturidade dos órgãos e sistemas fisiológicos^(2)^.

Dada a magnitude do problema, duas metas dos Objetivos de Desenvolvimento Sustentável (ODS) atentam para a reversão desse quadro. A meta 3 propõe acabar com os óbitos por causas evitáveis até 2030 nessa faixa etária^(1)^, o que é possível com cuidados perinatais que garantam melhores condições de nascimento. A meta 4 tem como foco central a garantia de acesso a um desenvolvimento infantil de qualidade^(4)^, o que implica oferecer cuidados e oportunidades que vão além da sobrevivência.

Uma especificidade da atenção ao bebê pré-termo é o atendimento por diversos profissionais de saúde. Esse atendimento geralmente é focado nos cuidados intensivos para a adaptação à vida extrauterina e pode ser mantido mesmo após a alta hospitalar, caso haja um risco clínico elevado ou o quadro de saúde da criança demande cuidados especiais diariamente^(5)^.

Nesse contexto de cuidados intensivos, os pais que acompanham o bebê durante a hospitalização relatam sentir-se inseguros diante dos cuidados que deverão prestar posteriormente. Segundo eles, a equipe de saúde não os prepara para a alta como gostariam, considerando todas as características da prematuridade^(6)^. Assim, ao longo da internação, é necessário o preparo da família para a alta, com intervenções que apoiem, orientem e, sobretudo, fortaleçam os pais para o cuidado domiciliar^(7)^.

Esse preparo é importante por dois motivos. O primeiro diz respeito à interação entre o bebê e a família, um dos aspectos centrais para o desenvolvimento infantil^(8)^. O segundo refere-se à insegurança dos pais quanto aos cuidados com o bebê, pois a falta de preparo durante a hospitalização para a transição ao domicílio fragiliza ações favoráveis ao desenvolvimento^(9)^.

A falta de orientação e apoio no cuidado transicional, relatada por pais de lactentes pré-termo, pode prejudicar o desenvolvimento dessas crianças, por estarem expostas a ambientes menos estimulantes^(10)^. Além disso, o desenvolvimento da criança é frequentemente pouco acompanhado nos serviços de saúde, que priorizam aspectos de crescimento, como o ganho de peso e estatura, em detrimento das habilidades funcionais e dos cuidados voltados à estimulação^(11)^. Por isso, a transição do cuidado hospitalar para o domiciliar configura um período crítico de adaptação e reorganização do cotidiano das famílias para atender às necessidades integrais desse bebê, indo além da preocupação com os dados antropométricos e sinais de doença^(12)^.

Com o intuito de auxiliar os pais nesse processo de transição e nos primeiros anos de vida, favorecendo o desenvolvimento infantil, foi criado um recurso educativo para famílias de crianças nascidas pré-termo: *História de Sofia: batalhas e conquistas da família no cuidado e desenvolvimento da criança prematura* ^(13)^. Trata-se de uma tecnologia educativa (TE), um livro, para facilitar o acesso a conhecimentos sobre cuidados e desenvolvimento infantil antes e após a alta hospitalar, ou seja, desde o ambiente da unidade de terapia intensiva. Ele contém orientações para o cuidado e a promoção do desenvolvimento, com textos simples, inúmeras ilustrações e um quadro sobre as habilidades funcionais — isto é, aquisições de mobilidade, interação social e autocuidado — para as idades de 0 a 3 anos e meio^(13)^.

Aborda a temática por meio da importância das interações e do afeto, explicitando como os processos de interação e cuidado, quando vivenciados em ambientes acolhedores e estimulantes, impulsionam o desenvolvimento da criança, com textos simples e inúmeras ilustrações^(13)^.

O cuidado promotor do desenvolvimento é vital para as crianças pré-termo, como já descrito^(6-10)^. Adicionalmente, investir no desenvolvimento infantil é uma estratégia eficiente para um país eliminar a extrema pobreza, promover o crescimento econômico inclusivo e ampliar a igualdade de oportunidades, razão pela qual essa temática integra os ODS^(14)^.

Assim, a pergunta deste estudo foi: Quais são os efeitos da TE História de Sofia sobre a estimulação domiciliar de lactentes nascidos prematuramente? Quais outras variáveis influenciam a estimulação domiciliar de lactentes nascidos prematuramente?

Justifica-se a realização da investigação sobre os efeitos dessa tecnologia educativa dada a magnitude do impacto do nascimento prematuro no desenvolvimento infantil e as experiências de carência de orientações específicas no cuidado transicional, o que pode acarretar pouca ou inadequada estimulação dessas crianças no domicílio. Conhecer os efeitos da aplicação da tecnologia, juntamente com outras variáveis que podem influenciar o cuidado domiciliar no contexto da prematuridade, contribuirá para a validação dessa TE como recurso para o cuidado transicional.

## OBJETIVOS

- Avaliar a efetividade da tecnologia educativa “*História de Sofia: batalhas e conquistas da família no cuidado e desenvolvimento da criança prematura”* para a promoção de estímulos domiciliares para lactentes pré-termo.
- Verificar associações entre fatores familiares, demográficos e comportamentais e a promoção de estímulos domiciliares para lactentes pré-termo.

## MÉTODOS

### Aspectos éticos

O estudo foi autorizado pelos comitês de ética das instituições de ensino e de saúde, seguindo a Resolução nº 466/12 do Conselho Nacional de Saúde, e registrado na plataforma virtual do Registro Brasileiro de Ensaios Clínicos como identificador primário RBR-2SK64P. Além disso, seguiu as recomendações do CONSORT para garantir a transparência e precisão do relato dos resultados.

A intervenção utilizou uma tecnologia educativa que pode ser considerada confiável e segura na abordagem do tema, considerando a metodologia robusta adotada para seu desenvolvimento^(11)^ e a validação de seu conteúdo por profissionais e famílias (dados ainda em processo de publicação). Este estudo faz parte dos testes clínicos necessários para a avaliação da TE quanto a seus potenciais efeitos na melhoria dos cuidados familiares e na promoção do desenvolvimento infantil.

### Desenho, período e local do estudo

Trata-se de um ensaio clínico pragmático (ECP) não randomizado, no qual a variável independente foi uma intervenção educativa, e o desfecho esperado foi a maior oferta de estímulos domiciliares para a promoção do desenvolvimento de lactentes nascidos pré-termo, especificamente no que se refere à interação da família com o bebê e às características e oportunidades do ambiente que favorecem o desenvolvimento infantil^(7,8)^.

Os ECPs são conduzidos em condições menos rigorosas do que os ensaios clínicos explanatórios, pois têm como objetivo avaliar a efetividade de uma intervenção em contextos que reproduzem as condições da prática clínica. Dessa forma, permitem que o estudo se adapte às necessidades e características dos participantes. Outro aspecto relevante do desenho do estudo refere-se à verificação da efetividade da intervenção, definida como a avaliação dos efeitos de uma intervenção em uma amostra ou população específica, considerando condições não controladas, ou seja, no contexto do “mundo real”^(15)^.

A não randomização considerou a impossibilidade de formar os grupos simultaneamente, pois os familiares dos recém-nascidos têm grande contato entre si durante a hospitalização na unidade de terapia intensiva neonatal (UTIN). Assim, o grupo controle (GC) foi formado pelas famílias cujos bebês já haviam recebido alta no início da coleta de dados, e o grupo experimental (GE), pelas famílias cujos bebês foram hospitalizados a partir dessa data (maio/2018). Tal estratégia garantiu que as famílias não tivessem oportunidade de troca de informações sobre a TE.

A coleta de dados foi realizada por meio de visita domiciliar a lactentes nascidos pré-termo em um hospital geral privado da zona leste da cidade de São Paulo, local em que a TE foi apresentada e ofertada.

### População, amostra e critérios de inclusão e exclusão

O estudo foi realizado com díades de pais e crianças nascidas no local do estudo. A amostra foi dimensionada para que a diferença padronizada entre os grupos nos escores do instrumento de avaliação fosse significativa em um teste estatístico de amostras independentes, definida como 80 díades, sendo 40 para o GC e 40 para o GE.

Para a formação do grupo controle, foi feito um levantamento no livro de admissão da UTIN, identificando-se os recém-nascidos pré-termo entre janeiro e maio de 2018, que já haviam recebido alta hospitalar. Para o grupo experimental, foram incluídos prospectivamente os recém-nascidos pré-termo que receberam alta hospitalar após maio de 2018. Ambos os grupos receberam as mesmas orientações padronizadas no serviço. Para o grupo experimental, adicionalmente, foi apresentada e entregue a TE. Após a oferta do livro, a pesquisadora solicitou autorização para contato telefônico em um período de 30 dias, para verificar o interesse em participar da pesquisa e realizar o agendamento da visita domiciliar.

Foram elegíveis para o estudo díades de pais e lactentes, com as seguintes condições: bebês nascidos com qualquer nível de prematuridade, independentemente de outras afecções; pais com condições cognitivas para realizar a leitura do material educativo e responsáveis pelo cuidado habitual da criança. Foram excluídas as díades com impossibilidade de agendamento da visita após três tentativas de contato via telefone, em horários alternados.

### Protocolo do estudo

A variável independente do estudo foi a exposição à tecnologia educativa no momento da orientação da alta hospitalar, e o desfecho foi a estimulação do lactente no ambiente domiciliar, mensurada pelo instrumento *Infant-Toddler Home*, ou *IT-HOME* ^(16)^.

O *IT-HOME* foi elaborado para medir, num contexto naturalista, a qualidade e a quantidade de estimulação e suporte disponíveis no ambiente doméstico para a criança, entendida como um receptor ativo de objetos, eventos e transações que ocorrem em conexão com o ambiente familiar^(17)^. É amplamente utilizado por pesquisadores em muitos países para avaliar os efeitos de intervenções de parentalidade e promoção do desenvolvimento infantil, especialmente em crianças em situação de vulnerabilidade^(18)^. Ele é composto por 45 itens, observados ou declarados, em relação às características contextuais e organizacionais do ambiente doméstico que influenciam o desenvolvimento infantil, divididos em 6 subescalas^(16)^:

1. Responsividade verbal e emocional da mãe: verifica as interações comunicativas e afetivas entre o cuidador e a criança;
2. Ausência de restrição e punição: verifica as maneiras utilizadas pelo cuidador para disciplinar a criança;
3. Organização do ambiente físico e temporal: verifica a rotina da criança dentro do contexto familiar e quais espaços e objetos são destinados à criança;
4. Disponibilidade de materiais, brinquedos e jogos apropriados: verifica e relaciona, de acordo com a idade da criança, a variedade e os tipos de brinquedos que estão disponíveis;
5. Envolvimento dos pais com a criança: verifica a qualidade e quantidade de interação que a criança recebe do principal cuidador;
6. Oportunidade de variação na estimulação diária: verifica as oportunidades de interações sociais que são oferecidas à criança além do contato com a mãe.

Todos os itens de cada subescala são pontuados como SIM ou NÃO, de acordo com um manual que fornece explicação de cada item e alguns exemplos para pontuá-los. Maiores pontuações indicam um ambiente familiar mais enriquecido de estímulos, e menores pontuações, no quartil inferior, representam risco para o desenvolvimento da criança^(16,17)^. Logo, o instrumento foi escolhido por sua potência em demonstrar se as famílias do GE promoviam um ambiente mais favorável ao desenvolvimento infantil, quando comparadas às famílias do GC, tendo como hipótese que os resultados diferentes entre os grupos estariam associados ao acesso à TE.

A pesquisadora principal foi responsável pela aplicação do *IT-HOME*, por meio de observação e entrevista semiestruturada com o cuidador principal da criança. É necessária a presença do bebê para obter informações imediatas sobre os padrões de interação entre o cuidador e a criança. A avaliação completa dura aproximadamente uma hora.

A coleta de dados ocorreu por meio de visitas domiciliares agendadas previamente por telefone, no período de maio a novembro de 2018. Nessas visitas, a pesquisadora era acompanhada por um entre três assistentes. Os assistentes eram enfermeiros com experiência no cuidado de lactentes pré-termo e atuaram como observadores, conforme previsto pelo instrumento de coleta, tendo recebido treinamento sobre os parâmetros psicométricos do *IT-HOME* ^(16)^. Não houve cegamento dos pesquisadores e das famílias, dado que a pesquisadora principal participou de todas as coletas e foi também a pessoa que compartilhou a TE no momento da alta. Todos os participantes tiveram a oportunidade de acompanhar a leitura e assinaram o Termo de Consentimento Livre e Esclarecido.

Para coletar dados sobre os fatores familiares, demográficos e comportamentais, bem como características do lactente pré-termo que poderiam influenciar seu desenvolvimento, foi utilizado um roteiro de questões que abordaram planejamento da gestação, preparo para a chegada do bebê, realização de leituras sobre desenvolvimento infantil, relatos de aspectos positivos ou negativos em relação ao cuidado, profissão dos principais cuidadores e anos de estudo. Tais informações foram obtidas por meio das respostas às questões do roteiro, como: Houve preparo para a chegada do bebê?, com duas opções de resposta: Sim ou Não. Todas essas informações, principalmente as relacionadas ao preparo para a chegada do bebê, poderiam influenciar o resultado do *IT-HOME*, uma vez que potencializam conhecimentos mais acurados sobre as necessidades de cuidados.

Para verificar a homogeneidade entre os grupos, foram levantados dados sobre as características físicas e fisiológicas dos bebês ao nascimento, bem como encaminhamentos pós-alta. A seguir, foi aplicado o *IT-HOME*.

Os dados foram registrados durante as visitas domiciliares em instrumento impresso e, posteriormente, digitados em dupla entrada em planilhas do *Excel* ^®^ versão 2016.

### Análise dos resultados e estatística

Para responder à efetividade da intervenção com a TE *História de Sofia*, realizou-se a análise estatística para comparação dos grupos e para a associação dos escores do *IT-HOME* com as variáveis de interesse. Para comparação de variáveis numéricas, foram utilizados os testes *t*, Wilcoxon-Mann-Whitney, Brunner-Munzel e ANOVA, além do teste de Kruskal-Wallis nos casos de mais grupos. Para comparação de variáveis categóricas, foram utilizados os testes qui-quadrado, teste exato de Fisher e coeficiente de Spearman.

## RESULTADOS

O fluxograma das famílias participantes do estudo, segundo recrutamento, alocação nos grupos controle e experimental e aplicação dos instrumentos de coleta dos dados está na Figura 1.

**Figura 1:** Fluxograma das díades participantes do estudo, segundo recrutamento, alocação e aplicação dos instrumentos de coleta dos dados, São Paulo, São Paulo, Brasil, 2019

As visitas domiciliares duraram de 40 a 120 minutos, com média de 80 minutos para a maioria das famílias (n=52, 74,3%). O GC foi composto por 30 famílias, e o GE por 32; ao todo, os dois grupos foram compostos por 35 lactentes, pois incluíram lactentes gemelares. Optou-se por manter os gemelares, ainda que as características das variáveis familiares e ambientais fossem similares, pois, ao aplicar o instrumento de coleta, houve pontuações diferentes entre as crianças quanto à estimulação ambiental em ambos os grupos.

Todos os lactentes tinham como principal cuidador a mãe; a maioria (41 - 58,5%) tinha um segundo cuidador: o pai (32 - 78%), a avó (5 - 12,3%) ou babá (4 - 9,7%). As mães do GC tinham, em média, 15,8 anos de estudo, e o segundo cuidador, 13,3 anos; as do GE tinham 16 anos de estudo, e o segundo cuidador, 14,2 anos de estudo, em média. Em relação à área de formação dos principais cuidadores, a prevalência foi na área de ciências humanas, com 18 (51,4%) no GC e 17 (48,6%) no GE. Não houve diferença estatisticamente significativa entre os grupos.

Quanto à idade dos lactentes no momento da coleta de dados, houve diferença estatisticamente significativa entre os grupos (p<0,001 - teste de Wilcoxon-Mann-Whitney), sendo 3,2 meses a média da idade corrigida do GC e 1,26 meses no GE. Quanto às condições de nascimento dos lactentes, item considerado para homogeneidade entre os grupos, não houve diferenças (Tabela 1).

**Tabela 1:** Características físicas e fisiológicas dos recém-nascidos dos grupos controle (GC) e experimental (GE) ao nascimento, São Paulo, São Paulo, Brasil, 2019

	GC	GE	Valor de *p*
Condições de nascimento			
Idade Gestacional Média	34,4 semanas	3,4 semanas	0,715**
Apgar de primeiro minuto	7,7 pontos	7,8 pontos	0,290**
Apgar de cinco minutos	8,8 pontos	9,0 pontos	0,64**
Peso médio de nascimento	2,2 gramas	2,1 gramas	0,384***
Peso/Idade Gestacional			
AIG*	25 (71,2%)	27 (77,2%)	0,785****
PIG*	09 (25,7%)	08 (22,8%)	
GIG*	01 (2,8%)	0 (0,0%)	

* *AIG – adequado para a idade gestacional; PIG – pequeno para idade gestacional; GIG – Grande para a idade gestacional;*

** *Wilcoxon-Mann-Whitney test;*

*** *Two Sample t-test;*

**** *Fisher’s Exact Test.*

Em relação aos diagnósticos médicos dos RN ao longo da internação, a principal patologia foi icterícia, GC (26, 74,2%) e GE (28, 80%), sem diferenças significativas entre os grupos. Não houve diferenças entre os lactentes do GC e do GE quanto aos encaminhamentos pós-alta, indicando complexidade de cuidados semelhantes entre os grupos.

Quanto à aplicação do *IT-HOME*, o GE obteve maiores escores médios, tanto na pontuação total do instrumento, quanto na maioria das subescalas (Tabela 2), mostrando maior estimulação ambiental para os lactentes do grupo que teve acesso à TE.

**Tabela 2:** Distribuição da pontuação média dos escores do *IT-HOME* nos grupos controle (GC) e experimental (GE), São Paulo, São Paulo, Brasil, 2019

Subescalas (Variáveis)	Média da pontuação por subescala		Valor de *p*
	GC	GE	
Responsividade emocional e verbal da mãe	9,54	9,60	0,513*
Ausência de punição e restrição	4,46	5,03	0,005*
Organização do ambiente físico e temporal	4,37	4,40	0,836*
Disponibilidade de materiais de brinquedos e jogos apropriados	3,03	4,14	0,001*
Envolvimento materno com a criança	2,37	3,80	<0,001*
Oportunidade de variação na estimulação diária	2,80	3,26	0,025*
*IT-HOME* Total	26,57	30,23	<0,001**

* *Wilcoxon-Mann-Whitney test;*

** *Two Sample t-test.*

As características comportamentais familiares tiveram associação com os resultados do *IT-HOME*. Houve homogeneidade entre os grupos quanto a tais características, conforme descrito a seguir.

A maioria das famílias dos dois grupos relatou mais aspectos positivos do que negativos sobre os cuidados com o bebê (60 - 85,7%). Tais relatos tiveram associação significativa com a subescala Responsividade Emocional e Verbal da Mãe, com pontuação média de 9,8 e mediana de 10 pontos, enquanto mais relatos de aspectos negativos, ou tanto negativos quanto positivos (10 - 14,3%), apresentaram pontuação média de 8,2 e mediana de 8 pontos (valor p<0,01 – teste Brunner-Munzel). Esse mesmo dado também influenciou a pontuação total do instrumento *IT-HOME*, cuja média de pontos foi 28,8 e mediana 29, frente à pontuação média de 29 e mediana de 25, na vigência de relatos mais negativos (valor p=0,007 - teste *Two Sample t*).

O relato de leitura de materiais específicos sobre DI como um tipo de preparo para a chegada do bebê influenciou de forma estatisticamente significativa duas subescalas e a pontuação total do *IT-HOME*, e ocorreu em (16 - 22,8%) famílias, divididas entre os dois grupos. Na subescala Disponibilidade de Materiais, Brinquedos e Jogos Apropriados, a pontuação média das díades desse grupo foi de 4,31 e a mediana 4,5, contra a média de 3,37 e mediana de 3 na ausência de tais leituras (valor p=0,019 - teste Wilcoxon-Mann-Whitney). Na subescala Envolvimento Materno com a Criança, a pontuação média foi de 3,63 e mediana de 3,5, contra a média de 2,93 e mediana de 3 para os que não leram (valor p=0,043 - teste Wilcoxon-Mann-Whitney). A média da pontuação total do *IT-HOME* para as famílias com essa variável positiva foi de 30,1 e mediana de 31, contra a média de 27,8 e mediana de 28 para as que responderam negativamente (valor p= 0,029 - teste *Two Sample t*).

Apenas sete famílias (10%) realizaram curso preparatório para a chegada do bebê, 3 no GE e 4 no GC, e essa variável influenciou duas subescalas e a pontuação total do instrumento. Na subescala Disponibilidade de Materiais, Brinquedos e Jogos Apropriados, a pontuação média dessas díades foi de 4,71 e mediana de 5, contra a média de 3,46 e mediana de 3 para as díades cujos pais não realizaram cursos (63 - 90%) (valor p= 0,026 - teste Wilcoxon-Mann-Whitney). Na subescala Envolvimento Materno com a Criança, a pontuação média foi de 4,14 e mediana de 4, contra a média de 2,97 e mediana de 3 para as que não realizaram tais cursos (valor p= 0,026 - teste Wilcoxon-Mann-Whitney). No escore total do *IT-HOME*, a pontuação média de 31 e mediana de 31 das díades cujas famílias participaram de cursos foi superior às demais, que obtiveram pontuação média de 28,1 e mediana de 28 (valor p= 0,05 - teste *Two Sample t*).

Utilizar a Unidade Básica de Saúde (UBS) foi apontado por 52 famílias (74,2%) como suporte aos cuidados com o lactente, e essa variável influenciou duas subescalas. Na subescala Disponibilidade de Materiais, Brinquedos e Jogos Apropriados, a pontuação média foi de 3,33 e mediana de 3, maior do que as famílias que não utilizavam esse recurso, cuja pontuação média foi de 2,39 e mediana de 3 (valor p=0,012 - teste Wilcoxon-Mann-Whitney). A subescala Envolvimento Materno com a Criança teve pontuação média de 3,33 e mediana de 3 nas famílias que utilizavam a UBS, e pontuação média de 2,39 e mediana de 3 entre as que não utilizavam esse serviço (valor p=0,027 - teste Wilcoxon-Mann-Whitney). Em relação à pontuação total do *IT-HOME*, as famílias que utilizavam a UBS tiveram pontuação média de 28,9 e mediana de 26, enquanto as demais apresentaram pontuação média de 26,7 e mediana de 29 (valor p= 0,027 - teste *Two Sample t*).

A maior parte das famílias, 41 (58,6%), também divididas similarmente entre os grupos, afirmou realizar atividades prazerosas com o bebê. Essas apresentaram pontuações maiores na subescala Disponibilidade de Materiais, Brinquedos e Jogos Apropriados, com pontuação média de 3,39 e mediana de 3, contra a pontuação média de 2,39 e mediana de 3 entre as que não realizavam tais atividades (valor p=0,027 - teste Wilcoxon-Mann-Whitney). Realizar tais atividades com o lactente também influenciou a subescala Envolvimento Materno com a Criança, com pontuação média de 3,39 e mediana de 4, contra a pontuação média de 2,66 e mediana de 3 para as que responderam negativamente (valor p= 0,027 - teste Wilcoxon-Mann-Whitney). Houve diferença na média da pontuação total do *IT-HOME* entre as famílias que realizavam atividades prazerosas com o bebê, com pontuação média de 29,32 e mediana de 27,1, enquanto as demais apresentaram pontuação média de 27,1 e mediana de 26 (valor p= 0,027 - teste *Two Sample t*).

As subescalas que tiveram mais influências de variáveis comportamentais familiares foram: Disponibilidade de Materiais, Brinquedos e Jogos Apropriados, com cinco variáveis associadas; Responsividade Emocional e Verbal da Mãe e Envolvimento Materno com a Criança, com três variáveis associadas a cada uma delas.

Adicionalmente às respostas aos objetivos estabelecidos, mais da metade dos participantes do GE (18 - 56,2%) emitiu comentários espontâneos sobre a TE, majoritariamente positivos: afirmaram que gostaram da TE (16 - 89%) e que a utilizavam como fonte de informação (14 - 78%).

## DISCUSSÃO

A TE mostrou-se efetiva para apoiar as famílias na oferta de um ambiente domiciliar com mais estímulos ao desenvolvimento infantil. A ausência de diferenças significativas entre os grupos quanto aos fatores demográficos e comportamentais prévios ao nascimento dos bebês, quanto às características físicas e fisiológicas dos recém-nascidos e quanto às variáveis pós-natais de uso de UBS e realização de atividades prazerosas com os bebês é sugestiva de que os efeitos observados são potencialmente associados à intervenção, que foi o acesso à TE.

Adicionalmente, a ausência de diferenças significativas nas variáveis ocupação, escolaridade dos pais, principal cuidador do bebê, características de nascimento, diagnósticos médicos e encaminhamentos pós-alta dos lactentes entre os grupos mostrou que a estratégia adotada para compô-los possibilitou formar grupos comparáveis.

A variável idade corrigida foi a única que apresentou diferença entre os grupos, com média maior no GC, o que poderia levar a resultados mais favoráveis para esse grupo. Essa afirmação sustenta-se no conhecimento de que as interações com os lactentes aumentam à medida que eles crescem e permanecem acordados por mais tempo^(19)^. Ao final do terceiro mês, idade já alcançada pelos bebês do GC, o lactente tem maior capacidade de resposta e de contato social^(20)^, o que poderia ter levado a maior estimulação nesse grupo, mas não ocorreu. Assim, os maiores escores de estimulação ambiental podem, de fato, estar associados a uma melhor compreensão das famílias acerca de sua importância, corroborando para o efeito positivo da TE.

A disponibilidade e variedade de brinquedos adequados à idade no ambiente dos lactentes foi maior no GE em relação ao GC, aspecto amplamente abordado na TE^(13)^. Ambientes que oferecem brinquedos ao bebê tornam-se atraentes para a aprendizagem e impulsionam o desenvolvimento^(21)^.

A TE tem um capítulo, *“Uma conversa sobre o incrível mundo do desenvolvimento”*, que destaca a importância dos brinquedos e como eles impulsionam o desenvolvimento, mesmo para essas crianças pré-termo e muito jovens^(13)^. Frequentemente, as famílias não consideram necessário brincar e interagir com o bebê nos primeiros meses de vida.

As práticas parentais e as interações sociais também são moduladores críticos para o desenvolvimento infantil^(22)^. O contato das famílias do GE com a TE estimulou essas interações, fazendo com que estivessem mais próximas de seus filhos e oferecessem oportunidades de interações sociais para além do contato com a mãe. Esses temas são ressaltados na TE, pois a interação por meio da conversa durante os cuidados rotineiros, bem como o contato com outras pessoas da família e da comunidade, é importante para o desenvolvimento infantil^(13)^. Por outro lado, os lactentes do GC recebiam menos estímulos ambientais, conforme a subescala Restrição e Punição do *IT-HOME*.

Uma parcela significativa das famílias demonstrou disponibilidade e envolvimento com o bebê desde antes do nascimento, por meio da realização de cursos preparatórios para o cuidado futuro e da leitura de materiais sobre desenvolvimento infantil, características que influenciaram positivamente os resultados do *IT-HOME*.

Essas características corroboram os achados de uma revisão que identificou os principais pontos discutidos na literatura sobre a influência da qualidade do contexto ambiental no desenvolvimento infantil. Interações positivas do indivíduo em seu contexto, principalmente familiar, os cuidados parentais, a oferta de bens e serviços e os fatores intrínsecos a esse ambiente são a base para o desenvolvimento infantil^(23)^.

Duas subescalas não apresentaram diferença significativa entre os grupos: a Responsividade Emocional e Verbal da Mãe e a Organização do Ambiente Físico e Temporal, sendo dados positivos na perspectiva de um bom cuidado em ambos os grupos. No caso da primeira, a maioria das famílias estava desenvolvendo ligação de afeto com seus filhos e, na segunda, pode estar associada à orientação de alta padronizada pelo serviço, pois os médicos recomendam aos pais evitarem passeios e a exposição do bebê em ambientes fechados com aglomeração de pessoas, pelo menos até ele atingir dois meses de alta ou obter liberação do pediatra.

Os resultados evidenciam que essa TE auxiliou as famílias no cuidado domiciliar ao lactente pré-termo, sendo, então, um recurso importante no preparo da alta hospitalar para a equipe de saúde. Esse achado corrobora ensaios clínicos com outras TE que são consideradas úteis para difundir conhecimento e informações e, sobretudo, facilitar a comunicação^(24,25)^.

A investigação de intervenções parentais em ambientes reais tem sido foco de atenção, sobretudo quando se trata da avaliação da efetividade de ferramentas que apoiam a promoção do desenvolvimento infantil^(26)^. Portanto, a TE *História de Sofia* pode ser considerada uma tecnologia efetiva para o alcance da meta 4.2 do ODS, pois possibilita aos pais de crianças nascidas prematuras oferecerem estímulos que promovem o desenvolvimento infantil com qualidade.

### Limitações do estudo

Como limitações do presente estudo, destacam-se a não randomização e o não cegamento das famílias participantes, bem como da pesquisadora, o que pode ter influenciado positivamente os resultados de algumas famílias sobre o desenvolvimento infantil. A não randomização foi justificada no método e garantiu a composição dos grupos; além disso, observou-se similaridade nas características individuais e familiares entre os grupos, o que pode ser considerado um atenuante. Quanto ao não cegamento, a presença dos observadores e a discussão dos registros da coleta de dados também contribuíram para minimizar a possibilidade de viés.

### Contribuições para a área da Enfermagem, Saúde ou Política Pública

O estudo apresenta a TE *História de Sofia* como uma importante ferramenta de apoio ao profissional da saúde durante o cuidado transicional, pois contribuiu para a ressignificação das práticas do cuidado, gerando autonomia para a promoção do desenvolvimento^(27)^ e reforçando a utilidade desse recurso para o alcance da meta 4.2 dos ODS.

## CONCLUSÕES

O contato com a TE pelas famílias de lactentes nascidos pré-termo durante o preparo da alta hospitalar levou a uma maior oferta de estímulos favoráveis ao desenvolvimento no ambiente domiciliar. Isso reforça a validade de ações educativas sistematizadas para as famílias dessas crianças, as quais apoiam a agenda dos Objetivos de Desenvolvimento Sustentável, especialmente no que diz respeito ao desenvolvimento infantil de qualidade na primeira infância.

Pesquisas futuras sobre a utilização da TE que façam o seguimento das díades e avaliem o desenvolvimento dos lactentes em idades mais avançadas poderão contribuir para conhecer melhor o alcance desse recurso.
